# Transcriptome and Biochemical Analysis Jointly Reveal the Effects of *Bacillus cereus* AR156 on Postharvest Strawberry Gray Mold and Fruit Quality

**DOI:** 10.3389/fpls.2021.700446

**Published:** 2021-08-09

**Authors:** Yi-Yang Yu, Guo-Xia Dou, Xing-Xing Sun, Lin Chen, Ying Zheng, Hong-Mei Xiao, Yun-Peng Wang, Hong-Yang Li, Jian-Hua Guo, Chun-Hao Jiang

**Affiliations:** ^1^Department of Plant Pathology, College of Plant Protection, Nanjing Agricultural University, Nanjing, China; ^2^Key Laboratory of Monitoring and Management of Crop Diseases and Pest Insects, Ministry of Agriculture, Nanjing, China; ^3^Engineering Center of Bioresource Pesticides in Jiangsu Province, Nanjing, China; ^4^Key Laboratory of Quality and Safety Risk Assessment in Agricultural Products Preservation (Nanjing), Ministry of Agriculture, College of Food Science and Technology, Nanjing Agricultural University, Nanjing, China; ^5^Jiangsu Coastal Area Institute of Agricultural Science, Yancheng, China; ^6^Jiangsu Provincial Key Construction Laboratory of Probiotics Preparation, College of Life Science and Food Engineering, Huaiyin Institute of Technology, Huai’an, China

**Keywords:** strawberry, gray mold, biological control, *Bacillus cereus* AR156, induced systemic resistance (ISR), transcriptome profiling

## Abstract

Postharvest strawberry is susceptible to gray mold disease caused by *Botrytis cinerea*, which seriously damage the storage capacity of fruits. Biological control has been implicated as an effective and safe method to suppress plant disease. The aim of this study is to evaluate the postharvest disease control ability of *Bacillus cereus* AR156 and explore the response of strawberry fruit to this biocontrol microorganism. *Bacillus cereus* AR156 treatment significantly suppressed gray mold disease and postponed the strawberry senescence during storage. The bacterium pretreatment remarkably enhanced the reactive oxygen-scavenging and defense-related activities of enzymes. The promotion on the expression of the encoding-genes was confirmed by quantitative real-time PCR (qRT-PCR) that significantly increased the expression of the marker genes of salicylic acid (SA) signaling pathway, such as *PR1*, *PR2*, and *PR5*, instead of that of the jasmonic acid (JA)/ethylene (ET) pathway, which was also shown. Moreover, through transcriptome profiling, about 6,781 differentially expressed genes (DEGS) in strawberry upon AR156 treatment were identified. The gene ontology (GO) classification and Kyoto Encyclopedia of Genes and Genomes (KEGG) pathway enrichment indicated that AR156 altered the transcription of numerous transcription factors and genes involved in the SA-related plant disease resistance, metabolism, and biosynthesis of benzoxazinoids and flavonoids. This study offered a non-antagonistic *Bacillus* as a method for postharvest strawberry storage and disease control, and further revealed that the biocontrol effects were arisen from the induction of host responses on the transcription level and subsequent resistance-related substance accumulation.

## Introduction

Strawberry (*Fragaria x ananassa* Duch.) is a popular fruit worldwide, and it is well known for its nutritional compositions and unique flavor. Postharvest strawberry easily suffers from infection of pathogenic microorganisms as well as the primary metabolism that leads to fruit rot and senescence, which finally results in the deterioration of fruit quality and waste of natural resources. The estimated loss of postharvest fruits is as high as 40–50% (Alkan and Fortes, 2015). Despite many other factors, fungal pathogens is the primary reason for the significant fruit losses during storage (Marín et al., 2017), and gray mold disease caused by *Botrytis cinerea* is the main fungal disease that threatens the storage of postharvest strawberries (Shao et al., 2013). Strawberries are frequently exposed to pathogenic microbes. Large doses of chemicals are required to prevent and control strawberry gray mold, which can easily lead to pathogen resistance and concerns on food safety and environment (Droby, 2005; Grabke and Stammler, 2015). How to safely and effectively control postharvest diseases and retard the decay has become an impending task.

Application of yeasts, filamentous fungi, and bacteria has become an effective means for the biocontrol of postharvest fungal diseases (Dukare et al., 2019). For instance, antagonistic yeasts are widely used for controlling postharvest diseases due to their simple nutritional requirements, extreme environmental tolerance, no toxic residues, and broad-spectrum of antibacterial properties (Spadaro and Gullino, 2010). *Bacillus* has been known as a safe and ecofriendly agent to control plant diseases. *Bacillus amyloliquefaciens* FZB42 has been shown to both repress the accumulation of tobacco mosaic virus and control lettuce bottom rot caused by *Rhizoctonia solani* by induced systemic resistance (ISR) (Chowdhury et al., 2015). The use of *Bacillus cereus* NRKT in vineyards contributed to the increase in the resveratrol content in berry skins by upregulating the gene expression of stilbene synthase, and is able to protect grape berries against fungal diseases (Aoki et al., 2017). In postharvest diseases, *Bacillus subtilis* CPA-8 inhibited the growth of fungal pathogens, including *B. cinerea*, *Monilinia laxa*, *Penicillium digitatum*, *in vitro*, and effectively reduced disease incidence of apple gray mold and stone fruit brown rot (Yánez-Mendizábal et al., 2011). *Bacillus pumilus* B19 reduced the size of gray mold lesion on apple caused by *Botrrytismali* (Jamalizadeh et al., 2010). *B*. *cereus* AR156 has also been reported to reduce the disease incidence of peach soft rot and loquat anthracnose rot, respectively, by inducing fruit resistance-related enzyme activities (Wang et al., 2013, 2014).

*Bacillus* control plant diseases through a variety of mechanisms, among which, the ability to activate ISR provides the host with long term and broad spectrum resistance to various diseases. Previously, it was found that *Pseudomonas fluorescens* WCS417r-triggered ISR-promoted plant resistance against the leaf pathogen, *Pseudomonas syringae* pv. *tomato* and the root pathogen, *Fusarium oxysporum* f. sp. *raphani* through jasmonic acid (JA) signaling pathway (Pieterse et al., 1998). Recently, Niu et al. (2011, 2012) showed that salicylic acid (SA) and JA/ethylene (ET)-dependent signaling pathways are both involved in *B. cereus* AR156-triggered resistance in *Arabidopsis thaliana* and tomato. Transcription factors (TFs), such as WRKY11 and WRKY70, also played an important role in the AR156-triggered ISR by regulating the transcription of resistance-related genes downstream of the two signaling pathways (Jiang et al., 2016b). The AR156 triggers plant immunity through host recognition of bacterial-secreted extracellular polysaccharides and other microbe-associated molecular patterns (MAMPs); however, the plant receptors for these MAMPs are still unclear (Jiang et al., 2016a). Studies on postharvest diseases have also revealed the remarkable role of defense-related enzymes in the process of microorganism-induced fruit disease resistance. The biocontrol yeast, *Hanseniaspora uvarum* reduced postharvest grape berry gray mold disease by increasing the activities of grape antioxidant enzymes, such as peroxidase (POD), superoxide dismutase (SOD), catalase (CAT), phenylalanine ammonia lyase (PAL), ascorbate peroxidase (APX), and polyphenoloxidase (PPO) (Cai et al., 2015).

In the recent years, with the advent of the post-genome era, omics technologies, such as transcriptomics, proteomics, and metabolomics, are experiencing rapid development. Transcriptome sequencing is a powerful tool for high-throughput research that helps reveal the differences in gene expression of the same organism in different growth periods and environments. Rao et al. (2019) combined targeted metabolome, the second-generation RNA sequencing (RNA-seq), and full-length transcriptome to explore the association between gene expression and polyphenol concentration in different developing stages of olive. Chen et al. (2019) analyzed the transcriptome of postharvest African Pride during cracking, and found that starch degradation and cell wall polysaccharide metabolism are closely related to fruit ripening and cracking. However, there are still few studies on its application in postharvest strawberry.

In this study, we found that *B. cereus* AR156 could improve the resistance of postharvest strawberry to gray mold and increase the storage capacity. In order to acquire a comprehensive understanding on the effect of AR156 treatment on strawberry fruit, we explored the resistance induced by AR156 from multiple perspectives. We first tested the effect of AR156 on the disease resistance and senescence-related enzyme activities of postharvest strawberry fruits. Quantitative real-time PCR (qRT-PCR) was used to verify the consistency of the expression of some of the defense-related enzyme encoding genes by promoting the enzyme activity and disease-resistant phenotype. Besides, to obtain more comprehensive information, we also identified 6781 differentially expressed genes (DEGs) induced by AR156 through comparative transcriptome, and the RNA-seq result was analyzed.

## Materials and Methods

### Plant Material and Strain Growth Condition

The strawberry (Benihoppe) used in this study was picked in Suoshi Ecological Garden, Jiangning District, Nanjing City, Jiangsu Province. Fruits with the same maturity and with no mechanical or pest damage were selected and shipped to the laboratory within 2 h. The *B. cereus* AR156, the plant growth-promoting rhizobacteria (PGPR) strain, was routinely cultured in Luria-Bertani medium (LB medium, 10 g of tryptone, 5 g of yeast extract, 10 g of NaCl per liter, and pH adjusted to 7.2). The strain was streaked out from –80°C freezer and incubated at 28°C. Single colony was picked out from the plate and inoculated into a shaking test tube with 5 ml LB for ∼16 h. The culture was then inoculated (1:100 v/v) into a flask with 500 ml LB medium. The culture was grown under shaking condition at 28°C for 24 h, and then centrifuged at 4°C, 5000 rpm for 10 min. Cells were collected and suspended with sterilized water to 5 × 10^7^ CFU⋅ml^–1^. *B. cinerea* BC1301 (Jiang et al., 2018a) was stored on potato dextrose agar (PDA, 200 g of potato, 18 g of glucose, 15 g of agar per liter) at 4°C. The strain was activated on PDA agar and cultured at 25°C for 7 days in dark. The mycelium was then scraped and dissolved in a sterile physiological saline (8.5 g/l NaCl). Spore was freshly prepared by filtering the sample against 8 layers of gauze and adjusted to 1 × 10^5^ spores⋅ml^–1^ using a hemocytometer.

### Antagonism Assay

The fungal mycelium stored at 4°C was placed on a PDA plate and cultured at 25°C. A sterile perforator was used to make fungal hyphae disk along the outer edge of the colony to obtain active growing hyphae. The fungal disk was transferred to the center of WA (5 g of peptone, 10 g of glucose, 3 g of beef extract, 5 g of NaCl, 15 g of agar per liter, pH was adjusted to 7.2) plate. Ten microliters of biocontrol bacteria suspension and water was dropped at the same distance from the fungal disk on the sterile filter paper disk. The antagonism plate was cultured in a 25°C incubator, and the growth of the fungus was observed. The antagonism diameter was recorded. This assay was repeated three times.

### Detection of Bacterial Effect on Gray Mold

Strawberry fruit was soaked in 0.001% sodium hypochlorite solution for 10 s and air-dried for 1 h. A 3 mm × 3 mm wound was created at the center of the fruit with a sterilized inoculating needle and air-dried. In the six AR156 treatments, the wound was treated with 50 μl 5 × 10^7^ CFU⋅ml^–1^ *B*. *cereus* AR156 cell suspension at 2 h, 6, 12, 18, 24, and 36 h, respectively, before inoculation of 50 μl 1 × 10^5^ spores⋅ml^–1^ *B. cinerea* spore suspension. The control was treated with 50 μl of water 24 h before inoculation with 50 μl 1 × 10^5^ spores⋅ml^–1^*B. cinerea* spore suspension. After treatment, the strawberry fruit was air-dried and placed in a container sealed with plastic film, and stored in an incubator at 20°C and relative humidity (RH) of 95%. The lesion diameter (in cm) on the strawberry fruit was observed every day and recorded whenever appropriate. Each treatment had three parallels, and each parallel contained 10 fruits. The experiment was repeated.

To verify the role of SA-signaling pathway in the process of disease suppression, the strawberry was sprayed with 0.3 mm 2-aminoindan-2-phosphonic acid (AIP) or paclobutrazol (PAC), respectively, before the wounds were created, and the samples were then air-dried after 1 h of incubation in room temperature. The disease control method is followed as above. *B*. *cereus* AR156 cell suspension was applied 24 h before the inoculation of the pathogen.

### Detection of Bacterial Effect on the Quality of Strawberry Fruit Stored at Low Temperature

Strawberry fruits with the same maturity and intact surface were selected and randomly divided into two groups of 300 each. Each group is then evenly divided into three parallels. Treatment group was evenly sprayed with 5 × 10^7^ CFU⋅ml^–1^ AR156 cell suspension (containing 0.001% Tween 20); control group was treated with 0.001% Tween 20 water solution. The surface of strawberry was air-dried. Samples were then placed in plastic baskets, covered with modified atmosphere bags to ensure internal air circulation, stored in a cold storage at 2 ± 1°C, and examined every 3–5 days to detect the quality-related indicators of the fruit. The specific methods are described in the sections below.

#### Measurement of Soluble Solids

A WYT-4 handheld sugar meter (Shanghai Precision & Scientific Instrument Co., Ltd., Shanghai, China) was used for soluble solids measurement. Ten fruits were measured for each treatment.

#### Measurement of Fruit pH

Fruit samples measuring 2 cm were gently taken from the fruit with a knife and ground. Samples were then removed to a flask, fixed to 100 ml of water, mixed thoroughly, and incubated for 20 min. The ground samples were filtered through four layers of gauze. Filtrate pH was measured at room temperature with a PHS-3C pH meter (Shanghai Precision & Scientific Instrument Co., Ltd., Shanghai, China). The test was repeated three times.

#### Measurement of Fruit Color

A Px.44-2132 colorimeter (Beijing Zhuochuan Electronic Science and Technology Co., Ltd., Beijing, China) was used to measure the surface color of fruit. The results were represented as L^∗^, a^∗^, and b^∗^, representing brightness, red and green, and yellow and blue, respectively. Symmetrical part at the center of the fruit was selected for measurement. Each treatment had three parallels, and each parallel contained 10 fruits.

#### Measurement of Vitamin C

Vitamin C was determined using an ultraviolet spectrophotometry (Nano Drop ND1000, Thermo Fisher Scientific, Inc., United States) following the Tillman’s method described by Santos et al. (2016) according to the following equations:

$$F=\mathrm{Mass}\mathrm{of}\mathrm{vitamin}C\mathrm{used}\mathrm{in}\mathrm{the}\mathrm{tritation}\left(\mathrm{mg}\right)/\mathrm{Volume}\mathrm{of}{\mathrm{Tillman}}^{\prime }s\mathrm{solution}\mathrm{used}\mathrm{in}\mathrm{titration}\left(\mathrm{ml}\right)$$

VitaminCcontent(mgper100ml)=V×F×100/A.

#### Measurement of the Relative Content of Fruit Flavonoids and Anthocyanins

Two grams of fruit tissue was ground thoroughly in 2 ml of 1% pre-chilled HCL–methanol solution. Samples were washed with 5 ml of HCL–methanol solution, incubated on ice for 20 min, and centrifuged at 4°C, 10,000 rpm, for 10 min. The absorbance at 325, 530, and 600 nm, respectively, of the supernatant was measured. The absorbance value of each gram of tissue at a wavelength of 325 nm represented the relative content of flavonoids; the difference between the absorbance values at wavelengths of 530 and 600 nm, respectively, represented the relative content of anthocyanins (U):

U=(OD-530OD)600⋅g.-1

### Detection of Bacterial Effect on Defense-Related Enzymes in Strawberry Fruit

The wound on the strawberry was created as described earlier. The four treatments are listed below. (1) Control treatment: 50 μl sterilized H_2_O was inoculated to the wound, 24 h later, another 50 μl sterilized H_2_O was applied to the wound; (2) AR156 treatment: 50 μl of 5 × 10^7^ CFU⋅ml^–1^*B*. *cereus* AR156 culture dilution was inoculated to the wound, then 50 μl sterilized H_2_O was applied to the wound 24 h later; (3) *B. cinerea* treatment: 50 μl sterilized H_2_O was inoculated to the wound, 24 h later, 50 μl 1 × 10^5^ spores⋅ml^–1^ of *B. cinerea* spore suspension was applied to the wound; (4) AR156 + *B. cinerea* treatment: 50 μl of 5 × 10^7^ CFU⋅mL^–1^ *B*. *cereus* AR156 culture dilution was inoculated to the wound, 24 h later, 50 μl of 1 × 10^5^ spores⋅ml^–1^*B. cinerea* spore suspension was applied to the wound.

The strawberry fruit was air-dried and placed in a container sealed with a plastic film, and stored in an incubator at 20°C and RH of 95%. The sampling time was 0, 12, 24, 36, 48, 54, 72, and 96 h after inoculation with *B. cinerea*. The wound tissue was cut with a scalpel sterilized with ethanol, frozen in liquid nitrogen, and stored in –80°C ultra low temperature freezer. The crude enzyme extract was prepared following the method described before (Qin et al., 2017). The fruit sample was placed in a liquid nitrogen pre-cooled mortar and ground into powder. Two grams of sample powder was collected in a pre-cooled test tube, and 5 ml of 50 mmol⋅l^–1^ pH 7.8 phosphate extract buffer [containing 1% polyvinylpolypyrrolidone (PVPP)] was added twice. Samples were vortexed at 4°C, centrifuged at 10,000 rpm for 10 min, and the supernatant was then collected. Each treatment contained three parallels, and the test was repeated two times. The APX, SOD, CAT, PAL, POD, and PPO enzyme activity was measured as described by Wang L. et al. (2019).

### RNA Extraction and Gene Expression Analysis

The strawberry fruit was air-dried and placed in a container sealed with a plastic film, and stored in an incubator at 20°C and RH of 95%. The tissue was cut with a scalpel sterilized with ethanol, frozen in liquid nitrogen, and stored in –80°C ultra low temperature freezer. Strawberry fruit samples (0.1 g each), treated according to the requirements of each treatment, were collected and ground into powder in liquid nitrogen. Total RNA was extracted with 1 ml of TRIZOL reagent (Invitrogen, Dalian, China) (Rio et al., 2010) and genomic DNA was removed by treating with RNase-free DNase (Takara, Dalian, China). HiScriptTM Q Select RT SuperMix and 1 μg of total RNA (Vazyme, Nanjing, China) were used for RNA reverse transcription according to the protocol of the manufacturer (37°C/15 min, 85°C/5 s). A constitutively expressed gene (18S rRNA in *Fragaria* x *ananassa*) was the reference gene in the qRT-PCR analysis. The PCR system: 2.5 μl of 10 × RT-PCR Buffer, 2 μl of 2.5 mm dNTP, 0.5 μl of Forward Primer, 0.5 μl of reverse primer, 0.5 μl of 50 × ROX, 1 μl of complementary DNA (cDNA), 0.25 μl of r-Taq, 2.5 μl of 100 × SYBR, and RNase-free dH2O was used to bring the total volume to 25 μl. The amplification procedure: 95°C/3 min + 40 × (95°C/15 s + 6 0°C/20 s + 72°C/30 s) + 95°C/15 s + 55°C/1 min + 72°C/30 s. The experiment was repeated three times for each treatment. The primers used in this work are shown in Supplementary Table 1.

### RNA Sequencing and Functional Annotation

The RNA sequencing library of the four treatments was constructed. In the mock treatments, strawberry wound was made as described above and treated with 50 μl of sterile water. One day later, 50 μl of 1 × 10^5^ spores⋅ml^–1^ *B. cinerea* spore suspension was inoculated. Sample was collected at 0 and 24 h after pathogen treatment in Mock_*B. cinerea*_at 0 h and Mock_*B. cinerea*_at 24 h treatments, respectively. For AR156 treatment, the strawberry wound was first treated with 50 μl of 5 × 10^7^ CFU⋅ml^–1^*B*. *cereus* AR156 cell suspension followed by the same pathogen inoculation procedure in mock treatment. Samples were collected at 0 and 24 h after pathogen treatment in AR156_*B. cinerea*_at 0 h and AR156_*B. cinerea*_at 24 h treatments. Strawberry fruit transcriptome sequencing was completed in Beijing Genomics Institute (BGI), China, using the HiSeqTM 2500 platform. The result has been uploaded to the National Center for Biotechnology Information (NCBI) in FASTQ format (BioProject accessions: PRJNA643674; BioSample accessions: SAMN15423441, SAMN15423442, SAMN15423443, SAMN15423444, SAMN15423445, SAMN15423446, SAMN15423447, SAMN15423448, SAMN15423449, SAMN15423450, SAMN15423451, SAMN15423452).

The data were processed as described by Jiang et al. (2019). Briefly, the clean data were acquired by processing the raw reads by Illumina Pipeline Software, and the adaptors and low-quality reads (*Q* < 20) were removed using Perl scripts. *Denovo* assembly of the transcripts was performed by Trinity method. The summary of the transcriptome assembly can be found in the Supplementary Data Sheet 1. Unigenes were then produced by mapping the data back to the contigs of the clean reads. Unigenes with significant expression were searched against the non-redundant protein sequence database using the NCBI BlastX (*E*-value ≤ 10–5). The blast result was mapped to UniProt, from which GO terms were extracted. Unigenes were further searched against several databases, such as the Swiss-Prot, the KEGG pathway database, and the GO database to acquire the putative function annotation.

### Identification of DEGs, TFs, and Plant Disease Resistance Genes

Mapped fragments per kilobase per million (FPKM) was used to represent the expression level of the identified genes. The DESeq R package (1.10.1) was used to analyze the differential gene expression. The DEGs were identified as genes with a log-fold expression change which is greater than 2 or less than –2 using a quality control threshold of false discovery rates (FDR) < 0.001 and a high statistically significant value of *P* < 0.05. The GO enrichment was obtained by enriching and refining the GO annotation acquired above using the GOseq R package using the ELIM method and Kolmogorov–Smirnov test (Supplementary Data Sheet 2). The KEGG pathways analysis was performed in KOBAS 2.0 server and enriched by using in-house scripts according to Fisher’s exact test. TFs in strawberry fruit were identified by blasting all assembled unigenes against the plant TF database^1^ with a threshold *E*-value of 1e-06 (Supplementary Data Sheet 3). Plant resistance genes (PRGs) were identified by searching all assembled unigenes in PRG database^2^ (Supplementary Data Sheet 4).

### Data Analysis

The data were analyzed using the statistical software, SPSS 24.0 (IBM SPSS Inc., United States). Differences were compared using the least significant difference (LSD test) (Fisher’s protected least significant differences test); the difference of *P*-value < 0.05 was considered as statistically significant.

## Results

### *B. cereus* AR156-Controlled Postharvest Strawberry Gray Mold Caused by *B. cinerea* and Delay Fruit Senescence

**FIGURE 1:**
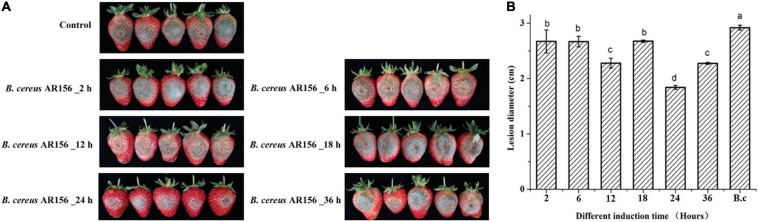
Effect of *B. cereus* AR156 on postharvest diseases of strawberry caused by *B. cinerea*. **(A)** Disease symptoms caused by *B. cinerea* infection of strawberry fruit with pretreatment of *B*. *cereus* AR156 at different times. **(B)** The lesion diameter of *B. cinerea* shown on strawberry fruit. Values were average numbers calculated from twelve different strawberry fruits after challenged with *B. cinerea*. All data were presented as means of three replicates ± SD, and error bars represented as SD for three replicates. Letters above the bars indicated statistically significant differences among treatments [least significant difference (LSD) test, *P* < 0.05]. All experiments were performed three times, and similar results were obtained.

The *B. cereus* AR156 has previously been shown to facilitate postharvest storage of peach and loquat fruit (Wang et al., 2013, 2014). At the starting point of this study, we wanted to investigate whether *B. cereus* AR156 has a positive effect on postharvest storage of strawberry fruits. In order to comprehensively understand the impact of AR156 on postharvest strawberry disease, we treated strawberry fruits with AR156 at different time points (2, 6, 12, 18, 24, and 36 h) prior to the inoculation of *B. cinerea* spore. Three days after pathogen treatment, the disease lesion diameter was recorded. The postharvest strawberry in the control treatment showed significant gray mold symptoms (Figure 1A) under room temperature storage (25°C). The lesion on strawberry fruits decreased significantly in the AR156 pretreatments (Figure 1B). Among all, compared with the control, the AR156 pretreated for 24 h had the most significant effect on disease lesion reduction, indicating the highest inhibitory effect on controlling the gray mold disease (Figures 1A,B). *B. cereus* AR156 was known to suppress fungal diseases through direct antagonism (Yu et al., 2017). We carried out an antagonism experiment on a plate to investigate whether it directly inhibits the growth of *B. cinerea*. The result showed that under laboratory condition, *B. cereus* AR156 had no significant ability to antagonize *B. cinerea* (Supplementary Figure 1).

**FIGURE 2:**
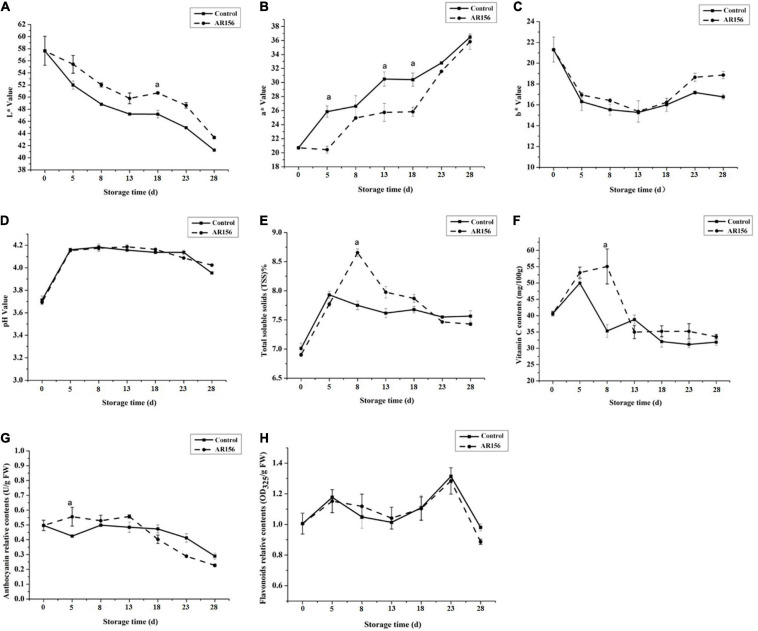
Effect of *B. cereus* AR156 treatment on the quality parameters of strawberry fruits during cold storage at 2 ± 1°C. The fruit quality of strawberry was evaluated in response to *B. cereus* AR156 treatment. Series of fruit quality parameters such as **(A)** L^∗^ value, **(B)** a^∗^ value, **(C)** b^∗^ value, **(D)** pH value, **(E)** total soluble solids, **(F)** vitamin C content, **(G)** anthocyanin relative contents, and **(H)** flavonoids relative contents were detected. All data were presented as means of three replicates ± SD, and error bars represent SD for three replicates. Letters above the bars indicate statistically significant differences among treatments [least significant difference (LSD) test, *P* < 0.05]. All experiments were performed three times, and similar results were obtained.

We also monitored the effect of AR156 on strawberry storage without pathogen stress by testing the fruit quality indicators. During storage, the L^∗^ value, a^∗^ value, and b^∗^ value of the strawberry underwent significant changes, while AR156 treatment suppressed this trend within the entire 28 days of test (Figures 2A–C). The total soluble solids and vitamin C content of the strawberry treated with AR156 were significantly improved compared to the treatment in the first 8 days, but reached consistency with the control in the later period (Figures 2E,F). However, the pH and relative contents of flavonoids and anthocyanins of strawberry fruit after AR156 treatment did not show significant difference from those of the control during the storage (Figures 2D,G,H).

### *B. cereus* AR156 Raise the Defense-Related Enzymes Activities of Strawberry by Inducing the Expression of Their Encoding Genes

**FIGURE 3:**
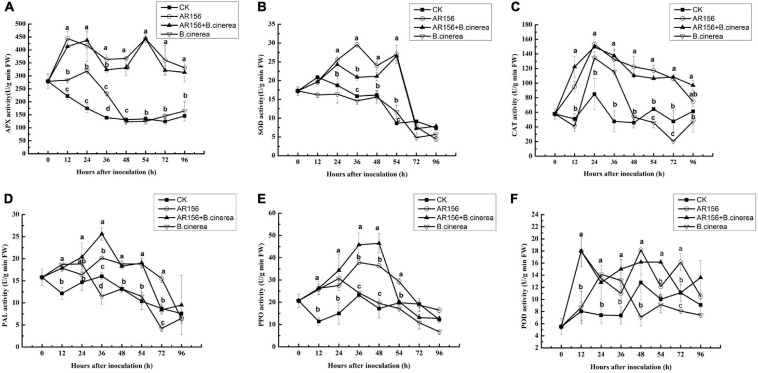
Effect on the activities of defense-related enzymes in the process of controlling postharvest diseases of strawberry by *B. cereus* AR156. Activities of defense-related enzymes in the strawberry pretreated with *B. cereus* AR156, and then were challenged with *B. cinerea*. **(A)** APX, **(B)** SOD, **(C)** CAT, **(D)** PAL, **(E)** PPO, and **(F)** POD. All data were presented as means of three replicates ± SD, and error bars represent SD for three replicates. Letters above the bars indicate statistically significant differences among treatments [least significant difference (LSD) test, *P* < 0.05]. All experiments were performed three times, and similar results were obtained.

To further explore the mechanism of the improvement of the beneficial bacteria on postharvest storage of strawberries, the effect of AR156 pretreatment on APX, SOD, CAT, PAL, POD, and PPO activities in the strawberry fruit with or without *B*. *cinerea* inoculation was tested. Pathogen treatment alone increased APX and CAT within 0–24 h. But the activities decreased rapidly afterward, and was lower than or equal to the control after 48 h. On the contrary, AR156 and AR156 + *B*. *cinerea* treatments could induce sustained enhancement on the activities of the both, within the tested 96 h (Figures 3A,C). After 48 h of storage, the APX enzyme activity in the AR156 + *B*. *cinerea* and AR156 treatment increased by 66.2 and 62.8% compared to the *B*. *cinerea* treatment, and the CAT activity increased by 56.2 and 51.5%, respectively. Similarly, AR156 and AR156 + *B*. *cinerea* treatments significantly induced the SOD activity of strawberry fruit than that of *B*. *cinerea* treatment and control from 24 to 54 h (Figure 3B). The AR156 rapidly increases the SOD activity in the early stage of infection. At 36 h, the SOD enzyme activity of the AR156 treatment reached the peak, which increased by 50.1% compared to the *B*. *cinerea* treatment. The activity of the SOD enzyme in the AR156 treatment showed a decreasing trend after 48 h of incubation; while the activity of the *B*. *cinerea* in the control treatment decreased during the entire testing period. *B*. *cinerea* induced the accumulation of PAL and PPO in the fruit soon after inoculation, but the significant difference from the control disappeared after 48 and 36 h, respectively. Instead, the activities of PAL and PPO, induced by AR156, reached the peak at 36 and 48 h, respectively, and were significantly higher than the control and *B*. *cinerea* treatment. The PPO enzyme activity in AR156 + *B*. *cinerea* and AR156 treatments was 61.6 and 56.2% higher compared to *B*. *cinerea* treatment at 48 h. Both, AR156 and *B*. *cinerea* could lead to a rapid increase on POD activity in the early stage, but AR156 treatment showed significant higher POD activity than the pathogen treatment and the control after 48 h.

**FIGURE 4:**
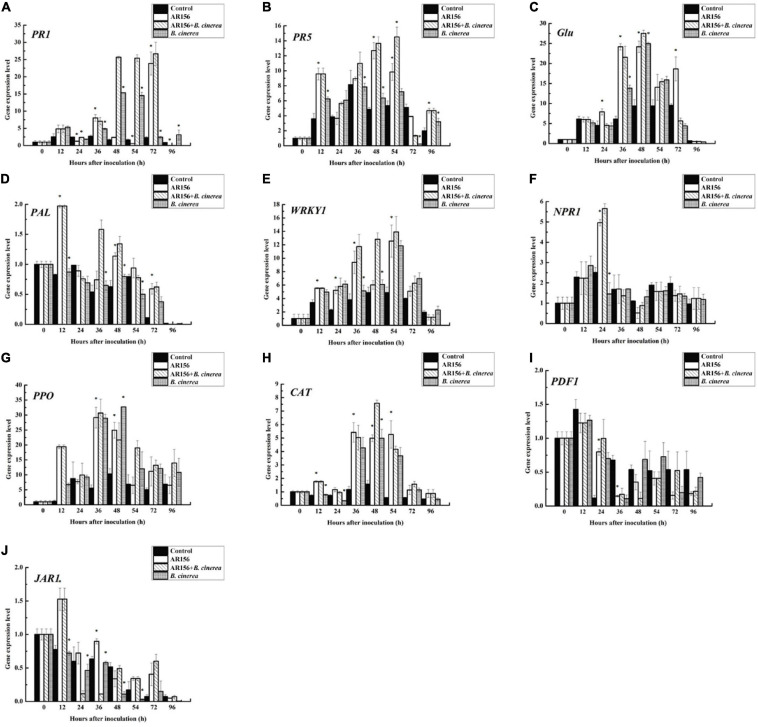
Effects on the expression profiles of defense-related gene in the process of controlling strawberry gray mold by *B. cereus* AR156. The expression of defense-related genes which were involved in SA and JA/ET signaling pathways in response to the interaction between *B. cereus* AR156 and *B. cinerea* were analyzed. Time course of the expression of these genes [**(A)** *PR1*, **(B)** *PR5*, **(C)** *Glu*, **(D)** *PAL*, **(E)** *WRKY1*, **(F)** *NPR1*, **(G)** *PPO*, **(H)** *CAT*, **(I)** *PDF1*, **(J)** *JAR1*] were detected in strawberry pretreated with *B. cereus* AR156 and challenged with *B. cinerea*. Asterisks indicate significant differences (*P* < 0.05) in gene expression caused by AR156 treatment under pathogen or non-pathogen conditions. The expression values of the individual genes were normalized using 18S rRNA coding gene as an internal standard. All experiments were performed three times, and similar results were obtained.

**FIGURE 5:**
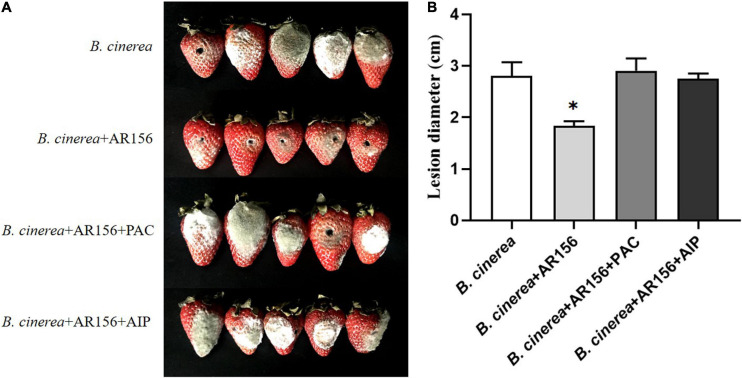
The effect of SA biosynthesis inhibitors on the control effect of AR156 against gray mold. **(A)** Disease symptoms caused by *B. cinerea* infection of strawberry fruit. *B. cereus* AR156 was pretreated 24 h before inoculation with the pathogen, and the strawberries were treated with 0.3 mm PAC or 0.3 mm AIP 1 h before AR156 treatment. **(B)** The lesion diameter of *B. cinerea* shown on the strawberry fruit. Values were average numbers calculated from 12 different strawberry fruits after challenged with *B. cinerea*. Bars were means ± SD. Asterisks indicate statistically significant differences between treatments (*P* < 0.05). The experiment was performed three times, and similar results were obtained.

It is rational to speculate that AR156 promoted the activity of these defense-related enzymes by altering the transcription of the coding genes. To verify this hypothesis, we extracted the RNA of strawberry fruits under different treatments at the above time points, and detected the expression of *PAL*, *PPO*, and *CAT*-encoding genes by qRT-PCR. The data showed that the AR156 and AR156 + *B. cinerea* treatments upregulated the expression of *PAL*, *PPO*, and *CAT* at 12 h compared to the control treatment, and the upregulation lasted for a long period during storage (Figure 4). We further tested the expression of other defense-related genes, and found that AR156 significantly induced the expression of SA signal pathway-related defense genes, such as *PR1*, *PR2* (*Glu*), *PR5*, while the expression of JA/ET signal pathway marker genes, such as *PDF1* and *JAR1* was not significantly affected. The AR156 and AR156 + *B. cinerea* treatment upregulated the expression of *PR5* at 12 h, and the expression of *PR1* and *PR2* was induced at 36 h compared to the control and *B. cinerea* treatment. *B. cinerea* treatment promoted the expression of *WRKY1* at 54 h, while AR156 + *B. cinerea* treatment was able to induce a stronger expression of *WRKY1* in advance by showing a significant difference on the expression in the control and *B. cinerea* treatments at 36 h. For *NPR1*, the AR156 and AR156 + *B. cinerea* treatments also upregulated the gene expression at 24 h. To further verify the role of SA-signaling pathway in the gray mold resistance induced by AR156, we treated the strawberry with the SA inhibitor, PAC/AIP, before AR156 treatment. The results showed that the control effect of AR156 against gray mold was impaired by PAC/AIP pretreatment (Figure 5).

### Identification and Functional Annotation of DEGs Upon *B. cereus* AR156 Treatment Revealed Induced Expression of Strawberry Defense-Related Genes in the Process of Strawberry Gray Mold Control

Based on the above results, we found that *B. cereus* could increase the accumulation of defense-related substances in strawberry fruits by modifying the gene transcription, thereby increasing the strawberry resistance to gray mold and slowing down the senescence of fruits during storage. In order to further understand how the genes and regulatory pathways were affected, we compared the transcription of strawberry fruit under AR156 or water treatment at 0 and 24 h after *B. cinerea* inoculation. The heatmap of Pearson’s correlation coefficients and principal component analysis (PCA) showed that the transcriptome repeats of each treatment had a high consistency. On the PCA analysis map, repeats of treatments tended to aggregate together, indicating the reliability of the transcriptome data (Supplementary Figure 2).

**FIGURE 6:**
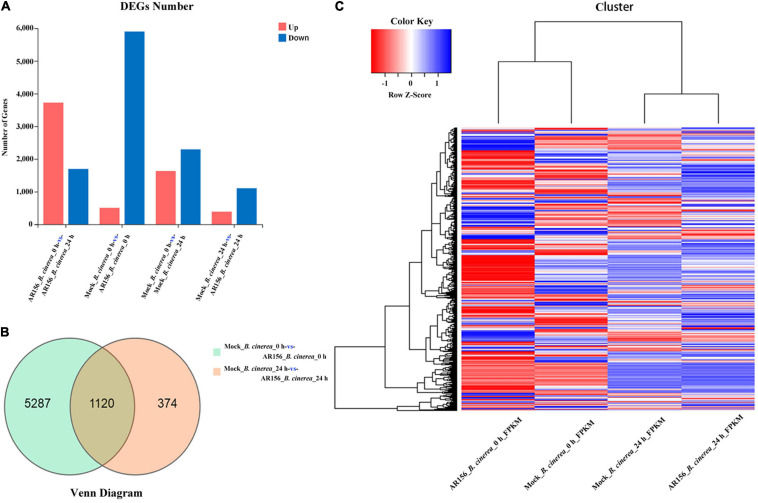
Classification of differentially expressed genes (DEGs) in the fruits of strawberry in response to *B. cereus* AR156 treatment and in combination with *B. cinerea* inoculation. **(A)** Numbers of DEGs in response to *B. cereus* AR156 treatment and in combination with *B. cinerea* inoculation. *X* axis represents DEG numbers *Y* axis represents comparison method between each group. Red color represents upregulated DEGs, blue color represents downregulated DEGs; **(B)** Venn diagram of DEGs in the fruits of strawberry treated by *B. cereus* AR156 treatment and in combination with *B. cinerea* inoculation at different time points; **(C)** Cluster analysis of DEGs in the fruits of strawberry treated by *B. cereus* AR156 treatment and in combination with *B. cinerea* inoculation at different time points based on the expression profiles measured by RNA sequencing (RNA-seq). The color scale in the heat map corresponds to log_2_^(FPKM)^ value of genes in each samples.

We analyzed the transcription sequencing data and identified DEGs as described in the section “Materials and Methods.” At the overall level of gene expression, the transcriptional profiles were consistent within each repeats (Supplementary Figure 3). By analyzing the differences among the treatments, we found that the expression of 6,781 genes, in the total of 45,353 genes identified in this study, was affected by the treatments. After 24 h of pathogen inoculation, AR156 could induce differential expression of 916 genes compared to all other treatments (Supplementary Figure 3). Further analysis of DEG revealed that 1,132 genes were upregulated in the AR156 treatment at 24 h compared to the control, and the number of downregulated genes was found to be 403 (Figures 6A,C). Comparing the AR156-caused DEG under pathogen and pathogen-free conditions, pretreatment with AR156 for 24 h led to a total of 6,407 DEGs, and the amount of DEGs caused by AR156 after pathogen inoculation was found to be 1,494 (Figure 6B). A total of 1,120 DEGs was induced by AR156 pretreatment at both 0 and 24 h, respectively, after pathogen inoculation, and the unique DEGs induced by AR156 was found to be 5,287 at 0 h, and 374 DEGs at 24 h. We also analyzed the magnitude of change in the gene expression. About 1,354 genes were upregulated at 24 h after pathogen inoculation by AR156 pretreatment. Among them, the expression of 124 genes was upregulated over 10-fold; 689 genes were downregulated, and the number of genes with over 10-fold expression was found to be 54 (Supplementary Figure 4).

### Pathway Enrichment Analysis for DEGs Upon *B. cereus* AR156 Treatment

For further investigation on the changes in the transcription of regulatory pathways and functional units in strawberry caused by AR156, we performed GO and KEGG pathway enrichment analyses with the above transcription data. In the GO analysis, the AR156 caused differential expression of genes, which was divided into three categories including biological process, cellular component, and molecular function. The DEGs were more concentrated in the cellular and metabolic processes under the classification of biological process, in the membrane part, cells, organelles, and membranes under cellular component and catalytic activity, binding under molecular function (Supplementary Figure 5). Through the enriched GO terms analysis, we found that DEGs were concentrated in intracellular and intracellular parts, cells, cell parts, organelles, and organelle parts (Supplementary Figure 5). The proportion of AR156-induced DEG of each category in the GO analysis was similar, regardless of the presence or absence of pathogen. However, the total number of DEGs under pathogenic condition was found to be 444 and 2,218 without the pathogen treatment. Interestingly, in the presence of *B. cinerea*, the AR156-induced DEGs were highly enriched in peptidase-related pathways, including endopeptidase inhibitor activity, peptidase inhibitor activity, endopeptidase regulator activity, peptidase regulator activity, serine-type endopeptidase inhibitor activity, sequence-specific binding, and response to stress pathway (Supplementary Figure 5).

In the KEGG pathway enrichment analysis, the AR156 induced DEGs were concentrated in the group of metabolites-related genes (Supplementary Figure 6). The enriched KEGG pathway shows that the DEGs induced by AR156 under pathogen-free condition were mainly enriched in ribosome-related genes. The AR156-induced DEGs in the presence of pathogen were mainly enriched in genes related to ribosome biogenesis, glyoxylate, and dicarboxylate metabolism. Moreover, the expression of benzoxazinoid biosynthesis and flavonoid biosynthesis-related genes has also been massively affected (Supplementary Figure 6). Further analysis showed that all three DEGs related to flavonoids biosynthesis were upregulated by AR156 treatment, regardless of the presence or absence of the pathogen. However, the effect of AR156 treatment on the gene expression was not consistent among the 16 identified DEGs related to benzoxazinoid biosynthesis (Supplementary Figure 7).

### Transcription Factors and Plant Disease Resistance Genes Prediction Based on DEGs Provided Potential Regulating Mechanisms of *B. cereus* AR156-Triggered Immunity

**FIGURE 7:**
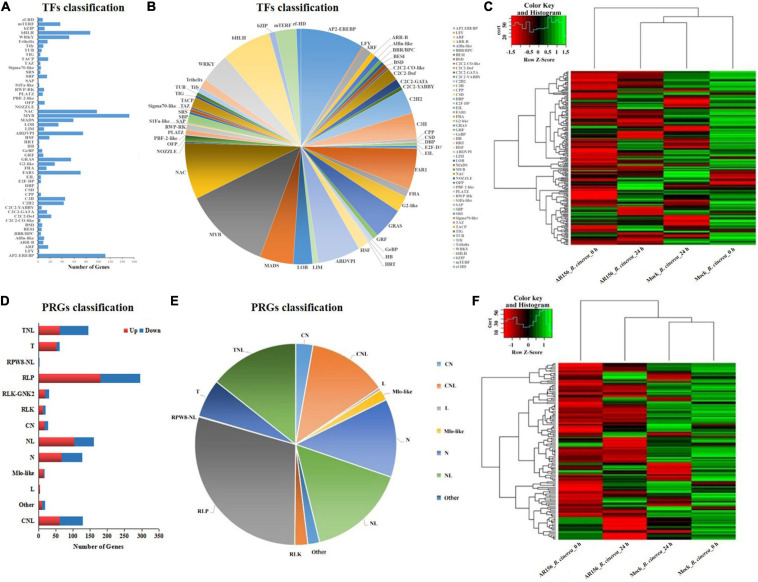
Transcription factors (TFs) and plant disease resistance genes (PRGs) prediction in the differentially expressed genes (DEGs) in response to *B. cereus* AR156 treatment and in combination with *B. cinerea* inoculation. **(A,B)** The classification of DEGs on TFs families in the comparisons of *B. cereus* AR156 pretreatment and mock treatment strawberry fruit, then both challenged with *B. cinerea*. In **(A)**, *X* axis represents the number of TFs, and *Y* axis represents TFs families; **(C)** Cluster analysis of identified differentially expressed TFs in the fruits of strawberry treated by *B. cereus* AR156 treatment and in combination with *B. cinerea* inoculation at different time points based on the expression profiles measured by RNA sequencing (RNA-seq). **(D,E)** Classification of DEGs on PRG families on comparing the strawberry fruits treated by *B. cereus* AR156 pretreatment and mock treatment, then both challenged with *B. cinerea*. In **(D)**, the *X* axis represents the number of PRGs, *Y* axis represents PRG families, and red and blue represent the number of upregulated and downregulated PRGs, respectively; **(F)** Cluster analysis of identified differentially expressed PRGs in the fruits of strawberry treated by *B. cereus* AR156 treatment and in combination with *B. cinerea* inoculation at different time points based on the expression profiles measured by RNA-seq. The color scale in the heat map corresponds to log_2_^(FPKM)^ value of genes in each sample.

Plants activate the transcript of a series of resistance-related genes in the process of resisting diseases, and the regulation of these genes often relies on the involvement of TFs. Therefore, we identified and analyzed TFs that were differentially expressed upon AR156 treatment. In this study, a total of 30 affected families of strawberry transcription factor were identified (Figure 7A), among which, NAC, WRKY, ERF, bHLH, and bZIP family transcription factors were widely regulated by *B*. *cereus* AR156 treatment (Figure 7B). Cluster analysis of identified differentially expressed TFs which showed that AR156 treatment could cause transcriptional change in many transcription factor encoding genes without disease infection (0 h after *B*. *cinerea* treatment) than in the presence of pathogen (24 h after *B*. *cinerea* treatment) (Figure 7C).

We analyzed the numbers and types of PRGs induced by AR156 in the samples. *B*. *cereus* AR156 could influence 12 families of PRGs, including CN, CNL, L, N, NL, RLK, RLK-GNK2, RPW8-NL, RLP, Mlo-like, T, and TNL, among which RLP was the most affected (Figures 7D,E). In nine of the 12 affected PRG families, there were more significantly upregulated PRGs than the down regulated ones (Figure 7D). In terms of the activation of disease-resistance gene transcription, the effect of AR156 treatment was more significant than that of the inoculation time (Figure 7F).

## Discussion

Postharvest strawberry diseases seriously threaten strawberry production. Biological control using beneficial microorganism is a safe method for postharvest disease control. In this study, we found that *B*. *cereus* AR156 could suppress strawberry gray mold (Figure 1). In addition, the application of AR156 significantly reduced the deterioration of the quality of the strawberry fruit during storage (Figure 2). *B*. *cereus* AR156 has been reported to control a variety of plant diseases, including rice sheath blight, root-knot nematode disease, and pepper *Ralstonia* wilt (Zhou et al., 2014; Yu et al., 2017; Jiang et al., 2018b). The ability of AR156 to directly antagonize a broad spectrum of pathogenic fungi, such as *R. solani* and *R. solanacearum*, contributes to the control of plant diseases (Yu et al., 2017; Wang N. et al., 2019). However, the result in this study showed that AR156 had no significant inhibition on the growth of *B. cinerea* (Supplementary Figure 1), which suggested that the control ability of AR156 against strawberry gray mold did not depend on bacterial antagonism. A series of *B*. *cereus* AR156 pretreatment at all sequential time points showed positive effect on the postharvest disease control. Among them, pretreatment of AR156 at 24 h before pathogen inoculation was the most effective in controlling strawberry gray mold (Figure 1). Based on the results, we hypothesized that *B*. *cereus* AR156 control postharvest disease by inducing strawberry resistance. The activation of defense may require time to allow the response of the activation of regulatory pathways and accumulation of resistance-related substances. The peak of the resistance induction effect in this study was at 24 h (Figure 1).

The quality of strawberry fruit consists of many factors. The color of the fruit directly affects its economic value. Soluble solids including sugar, acids, vitamins, and other nutrients, is an important nutrition and taste indicator of strawberries. During the storage of postharvest strawberry fruits, the AR156 treatment significantly inhibited the changes of strawberry color index, and improved the nutrition of the fruit (Figure 2). The changes in the endogenous nutrients of the fruit indicate that AR156 has a wide-ranging effect on the strawberry fruit. In fact, this is consistent with the large amount of DEGs induced by AR156 found under pathogen-free condition in this study. Among all the DEGs, we found that 73 and 668 genes were related to fruit development process and metabolic process, respectively; the differential expression of these genes may be responsible for the change in the storability of strawberry fruits (Supplementary Figure 5).

Induced systemic resistance is an important mechanism for beneficial microbes to assist the plants to fight against disease. There have been many reports showing the ability of *Bacillus* to enhance host immunity (Niu et al., 2012; Jiang et al., 2016b). APX, SOD, CAT, and POD are important defense enzymes for ROS metabolism in the plant (Gill and Tuteja, 2010). Damage caused by reactive oxygen species (ROS) is implicated in the process of senescence in the fruit (Tian et al., 2013). In this study, we found that AR156 treatment can significantly promote the enzyme activity of APX, SOD, CAT, and POD in strawberry fruit (Figure 3). The promoted enzyme activity coincides with the AR156-induced upregulated expression of coding gene (Figure 4H), indicating that AR156 enhances the activity of defense-related enzymes through transcriptional regulation. The increase of ROS metabolism-related enzyme activities may explain the ability of AR156 to retard the senescence of strawberry fruits under storage, which is consistent with the results of the fruit quality indicators (Figure 2). The PAL activity was also increased by *B*. *cereus* AR156 treatment even from the early stage of infection. It has been reported that the phenylalanine ammonium lyase (PAL) pathway is important for SA biosynthesis (Wildermuth et al., 2001). SA is a plant hormone widely involved in plant disease-resistance signal transduction, and PAL has been shown to be involved in plant systemic resistance and disease control through SA. For instance, it was found that acibenzolar-*S*-methyl (ASM) pretreatment of cucumber plant leaves primed the expression of a phenylalanine ammonia lyse homolog encoding gene, *PAL1*, which activates the SA pathway to protect the entire plant from *Colletotrichum orbiculare* (Cools and Ishii, 2002). The results of this study also showed that the expression of *PAL* gene in AR156-treated strawberry fruits was significantly higher than that of the control at 12 h (Figure 4). The changes of these defense-related enzyme activities were consistent with the induced genes expression, further indicating that AR156 affects the disease resistance of fruits by inducing resistance-related enzyme gene expression.

In addition to changes in defense enzymes, fungal infection often causes an accumulation of pathogenesis-related (PR) proteins. PR1, PR2 (Glu), PR5, and PR10 belong to the PR protein families, and are widely used as marker genes for the SA pathway. It has been shown that the infection of the bacterial pathogen, *Xanthomonas campestris* pv. *Vesicatoria*, led to the upregulation of *PR1* expression in pepper leaves (Jin Kim and Kook Hwang, 2000). In this study, *B*. *cereus* AR156 treatment effectively increased the expression of *PR1*, *PR2* (*Glu*), *PR5* in strawberry fruits from 12 h (Figure 4). The induced differential expression lasted at least 54 h, indicating that *B*. *cereus* AR156 could activate the early upregulation of PR gene expression, and the effect could be present for a long period. NPR1, as a key regulatory protein, controls the signal transduction of the SA-signaling pathway and is essential for activating the expression of PR genes. Overexpression of *AtNPR1* in citrus increases the resistance to citrus canker disease in an expression-depended manner (Zhang et al., 2010). The *NPR1* of strawberry was induced at 24 h after *B*. *cereus* AR156 inoculation (Figure 4), which suggested that the role of NPR1 in the resistance was induced by AR156 by regulating the expression of downstream *PR* genes. PAC and AIP are inhibitors of key enzymes in SA biosynthesis (Zon and Amrhein, 1992; Leon et al., 1995). We found that the ability of AR156 in disease suppression was impaired by the inhibitors treatment (Figure 5). Together, these results showed that *B*. *cereus* AR156 induced an early activation of the defense-related genes expression in strawberry mainly by activating the SA-signaling pathway in the process of controlling postharvest strawberry gray mold. The results of the GO pathway enrichment analysis showed that the AR156-induced biological processes related DEGs, regardless of the presence or absence of pathogens, mainly concentrated on cellular and metabolic processes (Supplementary Figure 5). This is consistent with the results of this study which states that AR156 could retard the senescence of strawberries as well as the previous reported ability of AR156 to prolong the storage time of peach and loquat fruits under cold storage conditions (Wang et al., 2013, 2014). For cellular component, AR156 can induce a large number of membrane-related DEGs (Supplementary Figure 5), which may be related to the pattern recognition of AR156 by the strawberry fruit. Plants can recognize MAMPs produced by beneficial microorganisms, thereby activating immune responses. The extracellular polysaccharide (EPS) produced by *B*. *cereus* AR156 has been reported as MAMPs with the ability to activate plant resistance to pathogens, but its pattern recognition receptor is still unknown (Jiang et al., 2016a). The membrane-related DEGs identified in this study may have the potential as a pattern recognition receptor, which remains to be tested. For the cellular component, AR156 could induce DEGs related to catalytic activity, which is consistent with the results that AR156 could induce a variety of defense-related enzyme activities (Figure 3). The KEGG pathway enrichment analysis showed that besides the metabolism-related DEGs, AR156 had a significant effect on the expression of genes relating to benzoxazinoid and flavonoid biosynthesis in the presence of pathogens (Supplementary Figure 6). The expression of all of the three DEGs related to flavonoids biosynthesis was upregulated by AR156 treatment (Supplementary Figure 7). Flavonoids are plant polyphenolic secondary metabolic compounds that protect plants from stress by functioning as signaling molecules and as antimicrobial agents. Benzoxazinoids protect plants from insect herbivores by acting as toxins and deterrents of feeding and digestibility. However, the biochemical test of AR156-treated strawberry showed no significant change in flavonoid content during cold storage (Figure 2). The discrepancy of gene expression and product may be due to a repressive effect on the metabolism of cold storage condition as the transcriptome sequencing sample was incubated under room temperature.

Transcription factors play key roles in regulating the plant defense against pathogen infection. This study analyzed the effect of AR156 treatment on strawberry transcription factor expression and found that the expression of a large number of MYB, NAC, WRKY, ERF, bHLH, and bZIP family transcription factors were affected. The WRKY family transcription factors are widely involved in the regulatory network of pathogen-induced cellular response processes in various plants. Many of the WRKYs participate in SA-mediated resistance and SA signaling pathways feedback regulation by activating or inhibiting SA responses (Chen et al., 2017). In a study using *Arabidopsis thaliana* as the host, the AR156 could induce the expression of resistance genes related to SA and JA/ET by regulating the expression of WRKY11 and WRKY70 (Jiang et al., 2016b). In consistent with the former study, *B*. *cereus* AR156 treatment quickly increased the expression of *WRKY1*, which was significantly higher during 36–48 h than in the pathogen treatment (Figure 4). The qRT-PCR results, together with the RNA-sequence data, provided evidence for the involvement of transcription factors in the AR156-induced postharvest strawberry disease resistance.

Based on the GO classification and functional annotation of DEGs, the prediction of strawberry resistance genes revealed an alteration on the expression of a large number of unknown PRGs upon AR156 treatment, suggesting the potential participation of PRGs in the AR156-induced disease resistance process. Further exploration of the functions of these unknown PRGs may help us to reveal more detailed mechanism of *B*. *cereus* AR156-induced disease resistance in strawberry fruits. Plants recognize MAMPs produced by beneficial microorganisms through pattern-recognition receptors (PRRs) to activate immune responses. MAMPs induce the transcription of receptor-like proteins (RLPs), such as LRR-RLKs, wall-associated kinases, and receptor protein kinase in *A*. *thaliana* (Qutob et al., 2006). We also found that upon AR156 treatment, more RLP-like DEGs were upregulated (180) than downregulated (116) (Figure 7). Some of these receptors have the potential to play a role in the recognition of AR156 by strawberries.

## Data Availability Statement

The original contributions presented in the study are publicly available. This data can be found here: National Center for Biotechnology Information (NCBI) in FASTQ format (BioProject accessions: PRJNA643674; BioSample accessions: SAMN15423441, SAMN15423442, SAMN15423443, SAMN15423444, SAMN15423445, SAMN15423446, SAMN15423447, SAMN15423448, SAMN15423449, SAMN15423450, SAMN15423451 and SAMN15423452).

## Author Contributions

Y-YY: writing the original draft, methodology, and data curation. G-XD: project administration, methodology, investigation, and data curation. X-XS: methodology and investigation. LC: investigation. YZ: methodology. H-MX: methodology and supervision. Y-PW: supervision and investigation. H-YL: investigation and writing – review and editing. J-HG: supervision, conceptualization, funding acquisition, and writing – review and editing. C-HJ: software, methodology, formal analysis, and writing – review and editing.

## Conflict of Interest

The authors declare that the research was conducted in the absence of any commercial or financial relationships that could be construed as a potential conflict of interest.

## Publisher’s Note

All claims expressed in this article are solely those of the authors and do not necessarily represent those of their affiliated organizations, or those of the publisher, the editors and the reviewers. Any product that may be evaluated in this article, or claim that may be made by its manufacturer, is not guaranteed or endorsed by the publisher.

## Footnotes

1
http://plntfdb.bio.uni-potsdam.de

2
http://prgdb.crg.eu/wiki/Main_Page
