# Supplementary material for: Transcriptome and Biochemical Analysis Jointly Reveal the Effects of Bacillus cereus AR156 on Postharvest Strawberry Gray Mold and Fruit Quality
Source: Front Plant Sci. 2021 Aug 9;12:700446. doi: 10.3389/fpls.2021.700446 (PMC8380966; doi:10.3389/fpls.2021.700446)
Supplement: Supplementary file 1 [file Data_Sheet_1.zip › Supplementary Tables and Figures.DOCX]

**Supplementary Table 1 Primers used in this study**

| Gene | Accession number | Forward primer ( 5’-3’) | Reverse primer (5’-3’) |
| --- | --- | --- | --- |
| *PR1* | AB462752.1 | ACATGGGATGCCAATCTAGC | CCACAGGTTCACAGCAGATG |
| *PR5* | － | CGTAGTTAGGTCCACCGAAGCATGTA | ACCTCCTAATGACACTCCCGAAACA |
| *Glu(PR2)* | AY989819 | TATAGATCAGTTTGTTATTTCTTCTTT | TGTTATCTGTAGAGCAGGCATAA |
| *NPR1* | － | ATCAGAAGCAACTTTGGAAGGTAGA | ACCGCCATAGTGGCTTGTTT |
| *WRKY1* | － | GATATGCGTTTCAGACCAGGAGCCAAGTCG | GCTTCTTCAGATTGCAACCCTGATGCGG |
| *PAL* | HM641823.1 | GTGAAAGAAGCGAAGAAGG | GAAGCTCGGAGCAGTATG |
| *JAR1* | － | TCTTCTGCTCAACATCAACATCGAC | CTACATGGCTTGAGAAATCAACAACTTC |
| *PDF1* | － | GCTTCTGAAGAGACGGTGATTCGCAGT | ATGGCTTGAAGCACATGCATTTTC |
| *CAT* | KC433883.1 | GTCTTCTTCGTCCGTGAT | GTAGTTCCCAGCAGCAAT |
| *PPO* | EU523113.1 | GGAGGCGGAGCAATAGAGAC | AGCCAGACCCCCTCAATACT |
| *18S rRNA* | X15590.1 | TGTGAAACTGCGAATGGCTCATTAA | GAAGTCGGGATTTGTTGCACGTATT |


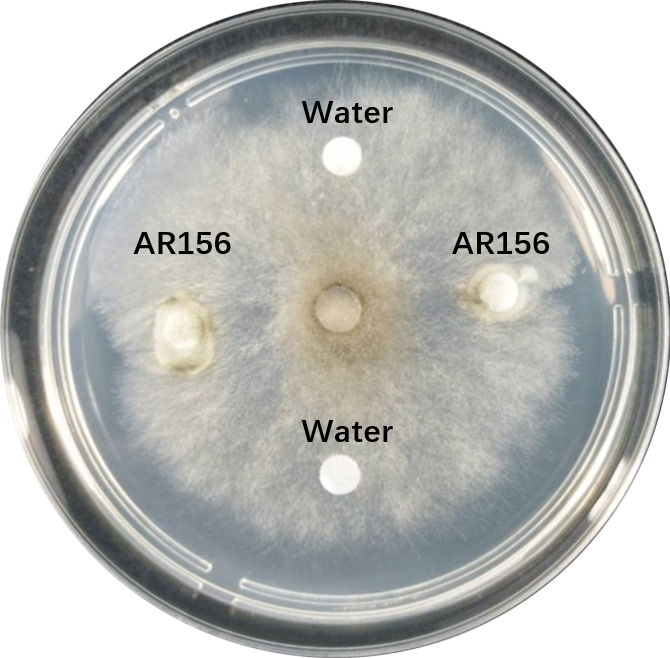


**Supplementary Figure 1 *In vitro* antagonism assay of *B. cereus* AR156 against *B. cinerea***

**
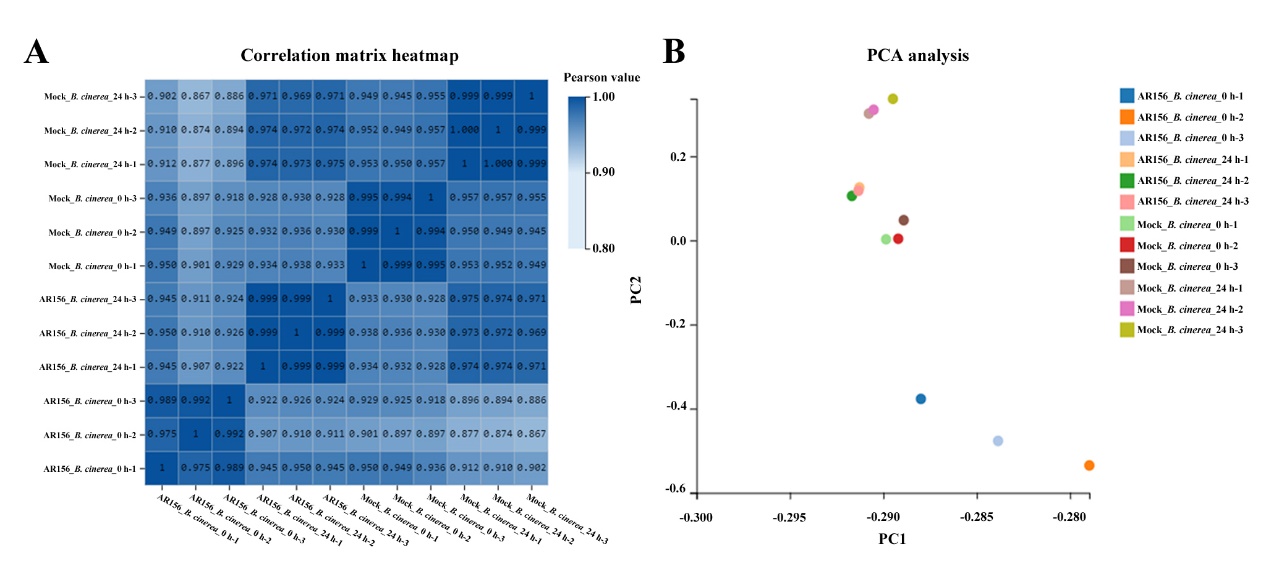
**

**Supplementary Figure 2 The correlation between different samples in the process of controlling strawberry gray mold by *B. cereus* AR156.**

Note: In order to reflect the gene expression correlation between samples, the Pearson correlation coefficients for all gene expression levels between each samples were calculated. A. Correlation analysis between samples and reflected in the form of heat maps. The X and Y axis represented each sample. The color represented the correlation coefficient (the darker the color, the higher the correlation); B. PCA analysis, X axis represented the contributor rate of first component. Y axis represented the contributor rate of second component. Points represented each sample. The samples in one group showed the same color.

**
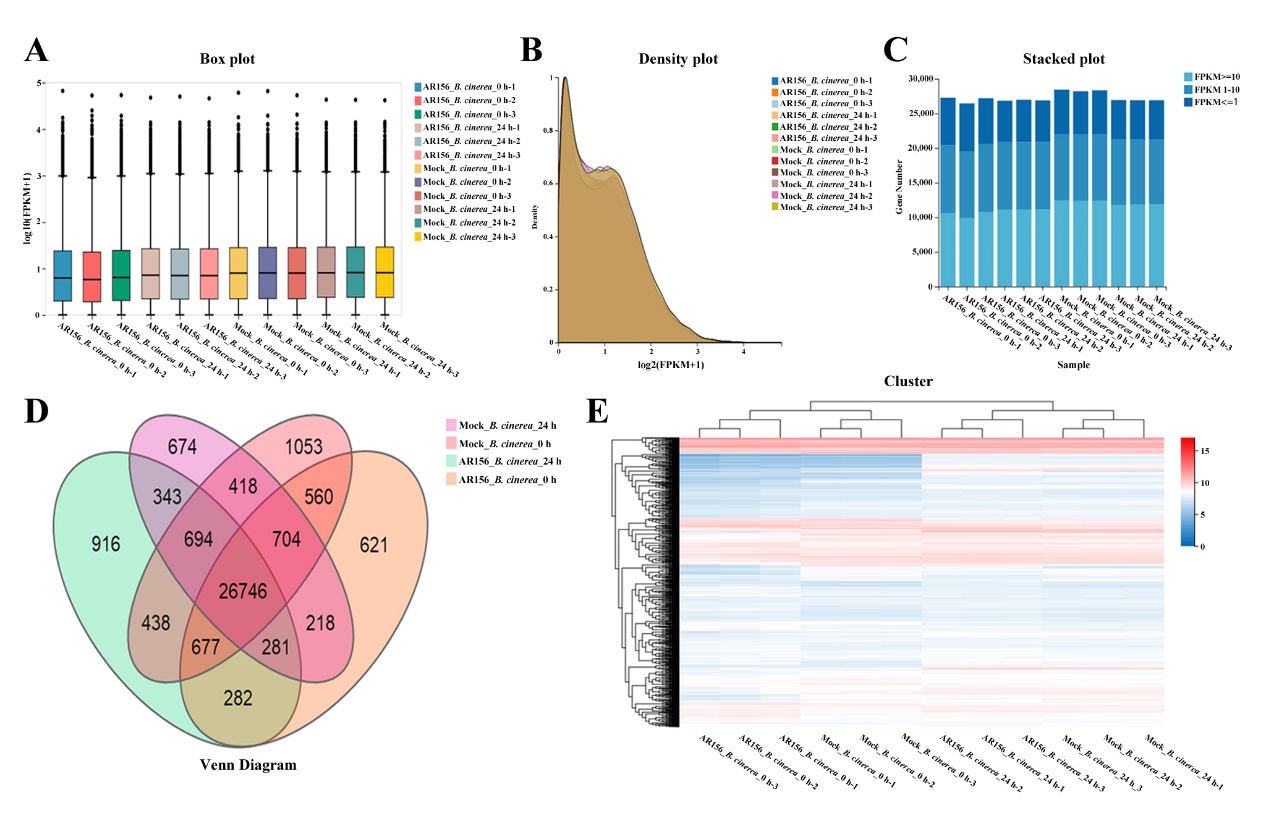
**

**Supplementary Figure 3 The distribution of gene expression from different samples in the process of controlling strawberry gray mold by *B. cereus* AR156.**

Note: A. Gene expression Box-plot. X axis represented the sample name. Y axis represented the log (FPKM) value; B. Gene expression density map. X axis represented the log (FPKM) value. Y axis represented the gene density; C. Gene expression distribution. X axis represented the sample name. Y axis represented the gene amount. The dark color meant the high expression level which FPKM value >= 10, while the light color meant the low expression level which FPKM value <= 1; D. Venn diagram of gene expression in the fruits of strawberry treated by *B. cereus* AR156 and challenge with *B. cinerea* at different time points; E. Cluster analysis of gene expression in the fruits of strawberry treated by *B. cereus* AR156 and challenged with *B. cinerea* based on the expression profiles measured by RNA-seq. The color scale in the heat map corresponded to log_2_ (FPKM) value of genes in each samples.

**
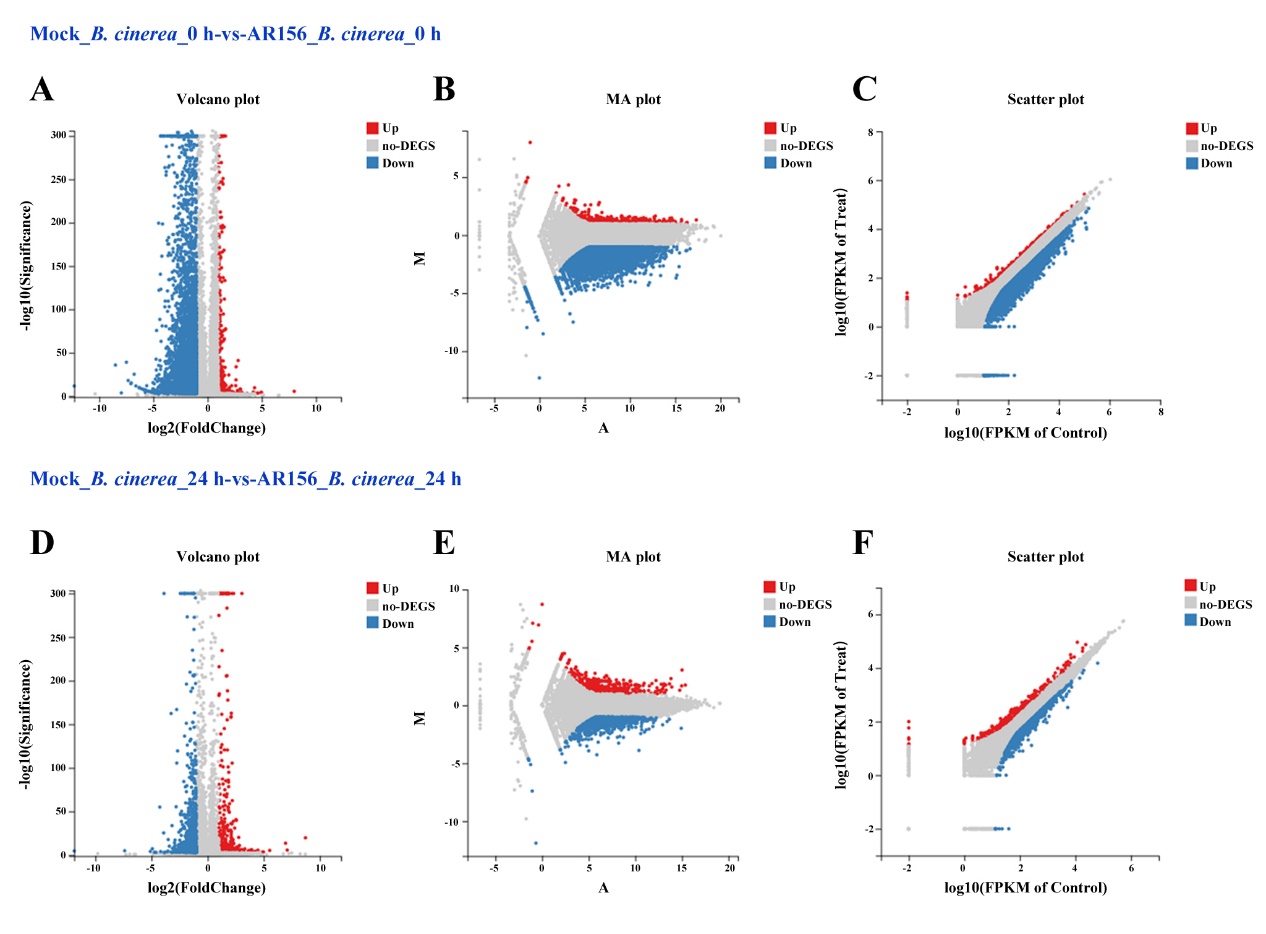
**

**Supplementary Figure 4 Distribution of DEGs identified from the fruits of strawberry in response to *B. cereus* AR156 treatment and in combination with *B. cinerea* inoculation.**

Note: A. and D. Volcano plot of DEGs. X axis represented log_2_ transformed fold change. Y axis represented –log transformed significance. Red points represented up-regulated DEGs. Blue points represented down-regulated DEGs. Gray points represented non-DEGs; B. and E. MA plot of DEGs. X axis represented value A (log_2_ transformed mean expression level). Y axis represented value M (log_2_ transformed fold change). Red dots represented up-regulated DEGs. Blue dots represented down-regulated DEGs. Gray points represented non-DEGs; C. and F. Scatter plot of DEGs. X Y axis represents log transformed gene expression level, red color represented the up-regulated genes, blue color represented the down-regulated genes, and gray color represented the non-DEGs. A-C showed the data from Mock_*B. cinerea*_0 h-vs-AR156_*B. cinerea*_0 h treatment, and D-F showed the data from Mock_*B. cinerea*_24 h-vs-AR156_*B. cinerea*_24 h treatment.

**
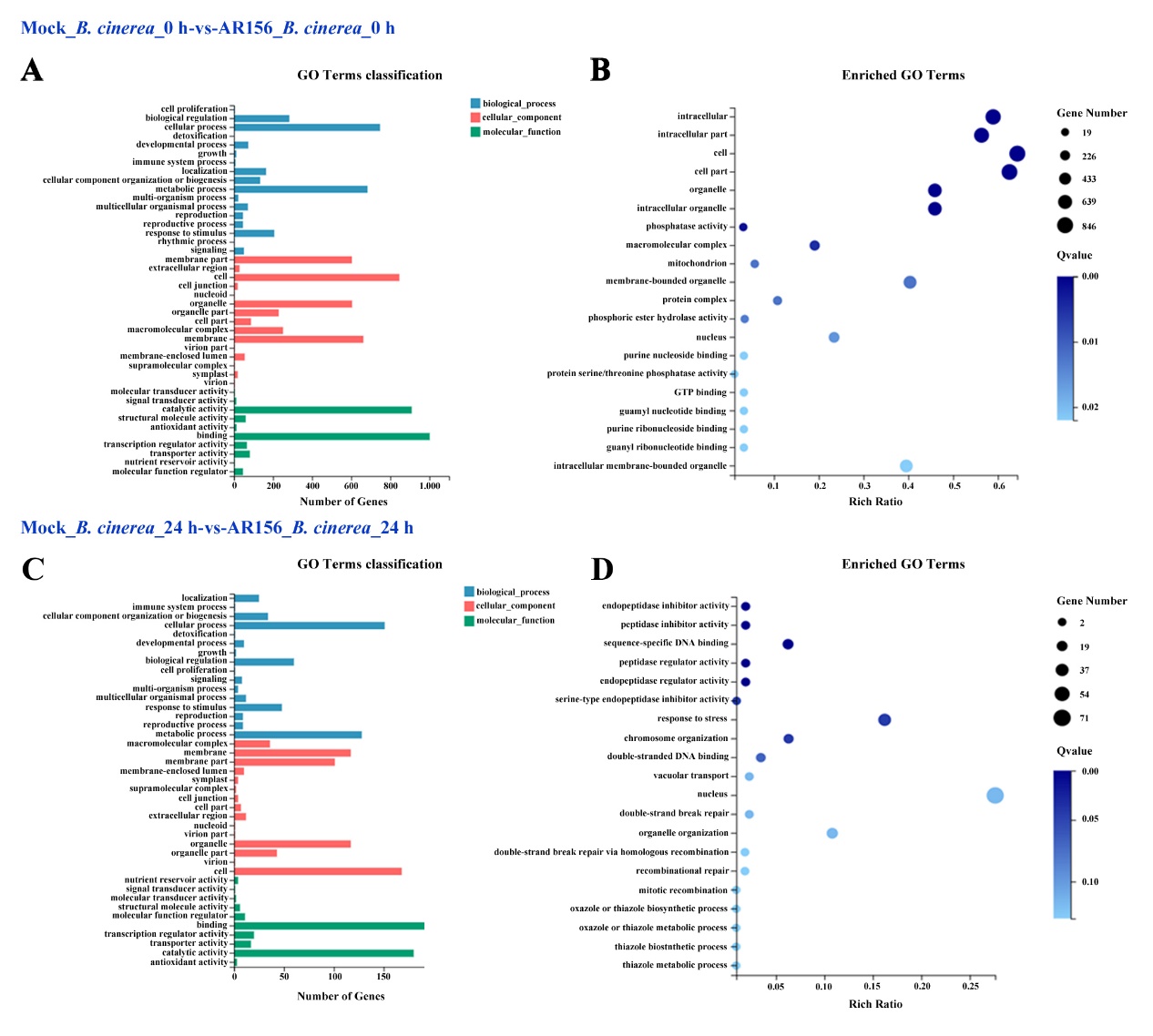
**

**Supplementary Figure 5 Gene Ontology (GO) analysis of up-regulated and down-regulated genes.**

Note: A. and C. GO classification of up-regulated and down-regulated genes in the comparisons of *B. cereus* AR156 pretreated or mock treated strawberry fruit, then both challenged with *B. cinerea* at different time points. X axis represented number of DEGs. Y axis represented terms classification of GO. There were three branches for GO terms: biological process, cellular component, and molecular function; B. and D. GO terms enrichment of DEGs. X axis represented enrichment factor. Y axis represented GO terms. The color indicated the q-value (high: white, low: blue), the lower q-value indicated the more significant enrichment. Point size indicated DEGs number (The bigger dots refer to larger amount). Rich factor referred to the value of enrichment factor, which was the quotient of foreground value (the number of DEGs) and background value (total Gene amount). The larger the value, the more significant enrichment. A-B showed the data from Mock_*B. cinerea*_0 h-vs-AR156_*B. cinerea*_0 h treatment, and C-D showed the data from Mock_*B. cinerea*_24 h-vs-AR156_*B. cinerea*_24 h treatment.

**
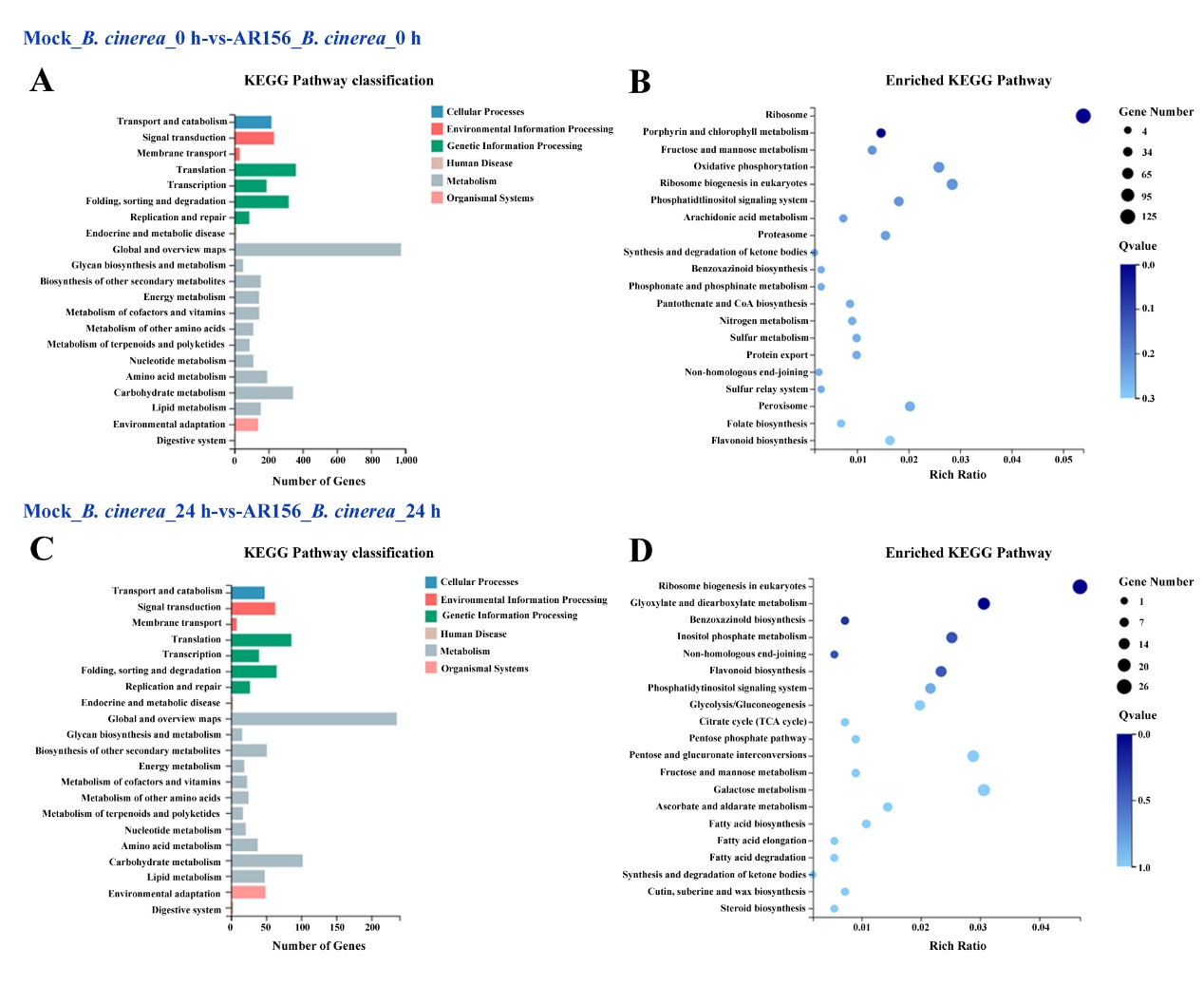
**

**Supplementary Figure 6 Kyoto Encyclopedia of Genes and Genomes (KEGG) pathway classification and functional enrichment of DEGs.**

Note: A. and C. Pathway classification of DEGs in the comparisons of *B. cereus* AR156 pretreated or mock treated strawberry fruit, then both challenged with *B. cinerea* at different time points. X axis represented number of DEGs. Y axis represented functional classification of KEGG. There were six branches for KEGG pathways: cellular processes, environmental information processing, genetic information processing, human disease, metabolism, and organismal systems; B. and D. Pathway functional enrichment of DEGs. X axis represented enrichment factor. Y axis represented pathway name. The color indicated the q-value (high: white, low: blue), the lower q-value indicated the more significant enrichment. Point size indicated DEGs number (The bigger dots referred to larger amount). Rich factor referred to the value of enrichment factor, which was the quotient of foreground value (the number of DEGs) and background value (total gene amount). The larger the value, the more significant enrichment. A-B showed the data in the Mock_*B. cinerea*_0 h-vs-AR156_*B. cinerea*_0 h treatment, and C-D showed the data in the Mock_*B. cinerea*_24 h-vs-AR156_*B. cinerea*_24 h treatment.


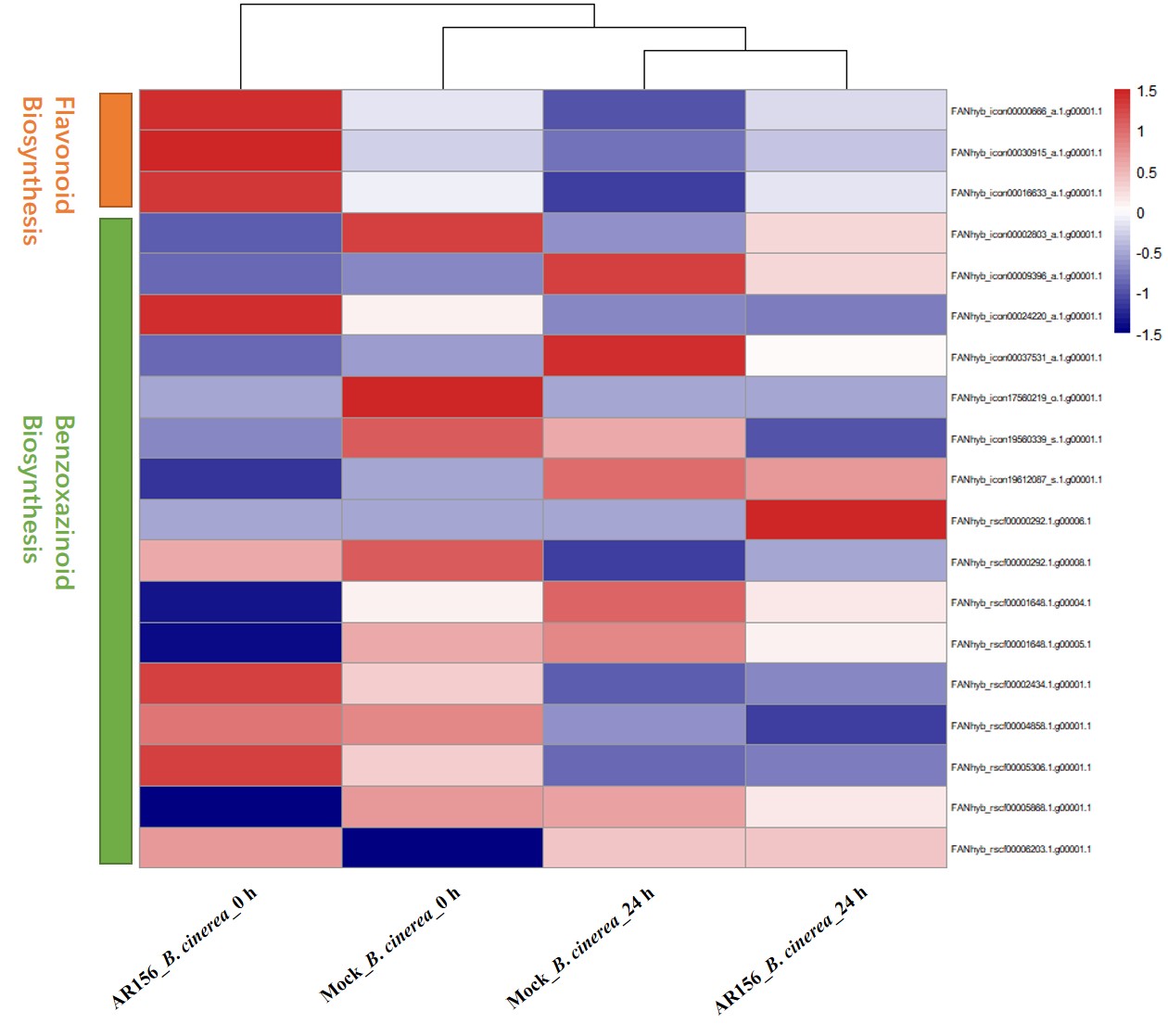


**Supplementary Figure 7 DEGs related to flavonoid and benzoxazinoid biosynthesis.**

Note: The heatmap showed the transcript abundance profiles of DEGs involved in flavonoid biosynthesis and benzoxazinoid biosynthesis under different treatments.
